# Arbuscular mycorrhizal fungi alter rhizosphere fungal community characteristics of *Acorus calamus* to improve Cr resistance

**DOI:** 10.7717/peerj.15681

**Published:** 2023-11-08

**Authors:** Guodong Xia, Sixi Zhu, Wei Zhao, Xiuqing Yang, Luying Sheng, Huan Mao

**Affiliations:** 1 Guizhou Minzu University, The Karst Environmental Geological Hazard Prevention of Key Laboratory of State Ethnic Affairs Commission, Guiyang, Guizhou, China; 2 Guizhou Minzu University, Guiyang, Guizhou, China

**Keywords:** Cr stress, Arbuscular mycorrhizal fungi, Fungal community structure, *Acorus calamus*, ITS rRNA sequencing technology, Phytoremediation

## Abstract

To investigate changes in fungal community characteristics under different Cr(VI) concentration stresses and the advantages of adding arbuscular mycorrhizal fungi (AMF), we used high throughput sequencing to characterize the fungal communities. Cr(VI) stress reduced rhizosphere soil SOM (soil organic matter) content and AMF addition improved this stress phenomenon. There were significant differences in fungal community changes under different Cr(VI) concentrations. The fungal community characteristics changed through inhibition of fungal metabolic ability, as fungal abundance increased after AMF addition, and the fungal diversity increased under high Cr(VI) concentration. The dominant phyla were members of the *Ascomycota, Basidiomycota, Mortierellomycota*, and* Rozellomycota*. Dominant groups relevant to Cr resistance were *Ascomycota* and *Basidiomycota* fungi. Moreover, Fungal community characteristics were analyzed using high-throughput sequencing of the cytochrome c metabolic pathway, NADH dehydrogenase, and NADH: ubiquinone reductase and all these functions were enhanced after AMF addition. Therefore, Cr(VI) stress significantly affects fungal community structure, while AMF addition could increase its SOM content, and metabolic capacity, and improve fungal community tolerance to Cr stress. This study contributed to the understanding response of rhizosphere fungal community in AMF-assisted wetland phytoremediation under Cr stress.

## Introduction

Nowadays, soil Cr contamination is a global concern (Li et al., 2019a), as Cr is a heavy metal that is harmful to plants and humans (Chen et al., 2018a; Chen et al., 2018b). It is often used in metallurgical industries, electroplating, dyeing agents and some alloy manufacturing (Dhal et al., 2013; Viti & Giovannetti, 2007; Manikandan et al., 2016). Naturally Cr is often found as stable compounds Cr(VI) and Cr(III) (Bartlett, 1991), with Cr(VI) mainly present in soil and water bodies as CrO_4_^2−^ and Cr_2_O_7_^2−^ (Xia et al., 2019). Cr(VI) is more toxic and more soluble than Cr(III), reported to be more than 100 times more toxic than Cr(III), and can cause DNA damage and carcinogenesis (Garcıa-Hernández, Villarreal-Chiu & Garza-González, 2017; Shi et al., 2018; Wang et al., 2017a; Wang et al., 2017b). Cr enrichment can have a severe impact on human health through the food chain. Therefore, it is crucial to remediate Cr(VI)-contaminated soils

Compared to physicochemical remediation, phytoremediation is a heavy metal remediation technique that has recently been identified as cheap and with no secondary contamination (Xue et al., 2018a; Xue et al., 2018b; Piyush, Singh & Anderson, 2020). Wetland plants, due to their well-developed root systems and vital growth capacity, play an indispensable role in the CWs (constructed wetlands) to treat wastewater containing heavy metals (Jan, Lerika & Jaroslav, 2009). However, studies have shown that high levels of heavy metal pollution can be toxic to wetland plants (Ammara & Saleh, 2020), and so to protect them, arbuscular mycorrhizal fungi (AMF) have been introduced (27 genera in one order, four families and 11 families in the phylum *Glomeromycota*, with about 300 species) (Redecker et al., 2013). AMF can form symbiotic relationships with the root systems of most terrestrial, aquatic and semi-aquatic plants (Brundrett & Tedersoo, 2018; Calheiros et al., 2019; Nataša & Gaberščik, 2010) which improves soil resources and responds effectively to environmental constraints (Balestrini et al., 2018; Lenoir, Fontaine & Sahraoui, 2016; Torres, Antolín & Goicoechea, 2018). AMF increases plant soil nutrient uptake by colonizing and altering their root systems, while at the same time improving their tolerance to heavy metals by increasing antioxidant enzyme (*e.g.*, catalase, peroxidase, and superoxide dismutase) activity and decreasing reactive oxygen species (ROS) (Lin et al., 2017; Wang et al., 2018; Devi, Gupta & Kapoor, 2019).

There have been numerous studies on bacterial stress to heavy metals (Fan et al., 2017; Sultana et al., 2014), and Cr(VI) reduction by bacteria (Wang et al., 2017a; Wang et al., 2017b; Xia et al., 2018; Viti et al., 2014; Xue et al., 2014), but few studies have addressed mechanisms of Cr(VI) reduction by fungi, especially heterotrophic fungi that are abundant in the soil/plant rhizosphere. Notably, most fungi have heavy metal chelating systems and heavy metal enrichment capabilities (Janoušková, Pavlíková & Vosátka, 2006; Aly, Debbab & Proksch, 2011). Fungal substrates for heavy metal resistance can be divided into intra- and extracellular (Hall, 2002). For example, some fungi can secrete organic acids and amino acids to chelate with heavy metals and thus reduce toxicity (intracellular; Vodnik et al., 2008), while others reduce heavy metal ions through electron transfer (extracellular; Xia et al., 2018). Cr(VI) removal by fungi studies are scarce, but some have shown that fungi also have antioxidant mechanisms to reduce Cr(VI) and thus reduce heavy metal toxic effects on themselves (Viti et al., 2014; Xue et al., 2014; Joutey et al., 2015; Acevedo-Aguilar et al., 2006). Fungal community structure is significantly altered in response to Cr stress (Del Val, Barea & Azcón-Aguilar, 1999; Nordgren, Baath & Soderstrom, 1983), however, few studies have investigated Cr(VI) fungal removal mechanisms and fungal community changes.

In this study, the aim was to compare fungal community structure and functional predictions in an AMF group with a control group using high-throughput sequencing, to reveal fungal response mechanisms after AMF addition under Cr stress, and also provide a basis for further studies on the principle of fungal reduction of Cr(VI). This study will also contribute to our understanding of the response of AMF-assisted wetland phytoremediation to Cr stress in inter-rhizosphere fungal communities. We hypothesized that the fungal community of *Acorus calamus* root soil would be affected in Cr-stressed soil. However, adding AMF could increase the stress of the fungi community to Cr by increasing SOM content.

## Materials and Methods

### Material selection

Pots used in the experiments were fully enclosed at the bottom, with bottom length, inner diameter, outer diameter and height of 13.5 cm, 19 cm, 21.5 cm and 14.8 cm, respectively, and were all sterilized. The plants used in this experiment were selected from *Acorus calamus*, which largely grows in constructed wetlands. Roots were disinfected with 75% alcohol and 1% sodium hypochlorite and then repeatedly rinsed with deionized water. Soil was collected using the five-point sampling method, to a depth of 20 cm, from the barren hills behind the Lost Wetland Research Centre, mixed well and taken back to the laboratory. Large weeds and stones were removed and the soil passed through a two mm sieve, sterilized and used as a culture substrate (pH =6.83). Three kilograms of soil was placed in each sterilized pot. Rhiaophagus intraradices (purchased from Changjiang University) was the AMF used Walker & Schuessler (2010), in a substrate: strain ratio of 5:1.

### Experimental design

Experimental groups were divided into AMF-added, and non-AMF-added, each with five Cr concentration levels (0, 10, 50, 100, 200 mg/kg), and three parallel groups for each treatment. Each experimental pot was disinfected with 75% ethanol and 1% sodium hypochlorite solution for 10 s and 15 mins and then carefully cleaned with deionized water five times (Hu et al., 2021). Seedlings were placed in the experimental pots, incubated at a constant temperature of 26 °C and a light intensity of 176 µmol m^2^ s^−1^ for a photoperiod of 12 h for 30 days. The greenhouse humidity was stable at 60%rh, then inoculated with AMF and observed after 15 days. After successful planting, the experiment was conducted by adding potassium dichromate in five concentration gradients, followed by Hogaland’s solution (15 mL) and water (150 mL) every 14 d and 3 d, respectively (Zhan et al., 2019).

### Sample analysis

At the end of the 30-day experiment, the inter-root soil under the uniformly growing yellow iris was removed, and passed naturally through a two mm sieve to determine soil pH, Electrical Conductivity (EC), SOM, total Cr and Cr(VI). Soil pH was determined by potentiometric titration, soil organic matter (SOM) by oxidation with K2Cr2O7-H2SO4—external heating, and total Cr and Cr(VI) in the soil by extraction with the alkaline solution—flame atomic absorption spectrophotometry method. Another portion of each soil sample was stored in a −20 °C freezer and used for DNA extraction and PCR amplification (Zhao et al., 2023).

### DNA extraction and PCR amplification

Total DNA extraction from the microbial community was carried out according to the E.Z.N.A.^®^ soil DNA kit (Omega Bio-Tek, Norcross, GA, U.S.) instructions, and DNA extraction quality was determined using 1% agarose gel electrophoresis. PCR amplification of the ITSrRNA gene was performed using ITS1F (5-CTTGGTCATTTAGAGGAAGTAA-3′) and ITS2R (5-GTTTGCGTTCTTCATCGATGC-3′), buffer 4 µL, 2.5 mM dNTPs 2 µL, upstream primer (5uM) 0.8 µL, downstream primer (5uM) 0.8 µL, TransStart FastPfu DNA polymerase 0.4 µL, template DNA 10 ng and ddH2O to 20 µL. 3 replicates per sample (Wang et al., 2021).

### Illumina Miseq sequencing

PCR products from the same sample were mixed and recovered on a 2% agarose gel. Recovered products were purified using the AxyPrep DNA Gel Extraction Kit (Axygen Biosciences, Union City, CA, USA), detected by electrophoresis on a 2% agarose gel, and analyzed with a Quantus™ Fluorometer (Promega). A fluorometer (Promega) was used to quantify the recovered product. Library construction was performed using the NEXTflexTM Rapid DNA-Seq Kit (PerkinElmer) by: (1) splice linkage; (2) removal of splice self-linked fragments using magnetic bead screening; (3) enrichment of library templates using PCR amplification; and (4) recovery of PCR products from magnetic beads to obtain the final library. Sequencing was performed using Illumina’s Miseq PE300 platform (Shanghai Meiji Biomedical Technology Co., Ltd., Shanghai, China) (Chen et al., 2016).

### Statistical analysis

Shapiro–Wilk and Levene’s tests were used to verify the normality and homogeneity of the data. Data were mean ± standard deviation for three replicates. Two-way ANOVA and Tukey’s HSD post were used to analyze the differences among treatments. When the assumptions of normal distribution and homogeneity of variance are not satisfied, the non-parametric Kruskal-Wallis test is used to analyze the data, plotting by Origin and GraphPad prism. Fungal species annotation and assessment using mother (version v.1.30.2) index analysis, and species composition was based on the R language (version 3.3.1; R Core Team, 2016) vegan data package. Samples Qiime was used to calculate beta diversity distance matrices in comparative analyses, followed by tree drawing in R (version 3.3.1; R Core Team, 2016). Environmental factor association analyses were analyzed and mapped using RDA in the R language vegan package, and associated heat maps were used in R (heatmap package). Based on the PICRUSt2 function prediction package, predictions were made against the ITS amplicon sequencing results to obtain KO, pathway, and EC information based on information from the KEGG database and to calculate the abundance of each functional class based on OTU abundance. Additionally, for Pathway, PICRUSt was applied to obtain information on the three levels of metabolic pathways, and the abundance of each level was obtained separately.

## Results

### Analysis of root/soil environmental factors

**Figure 1:**
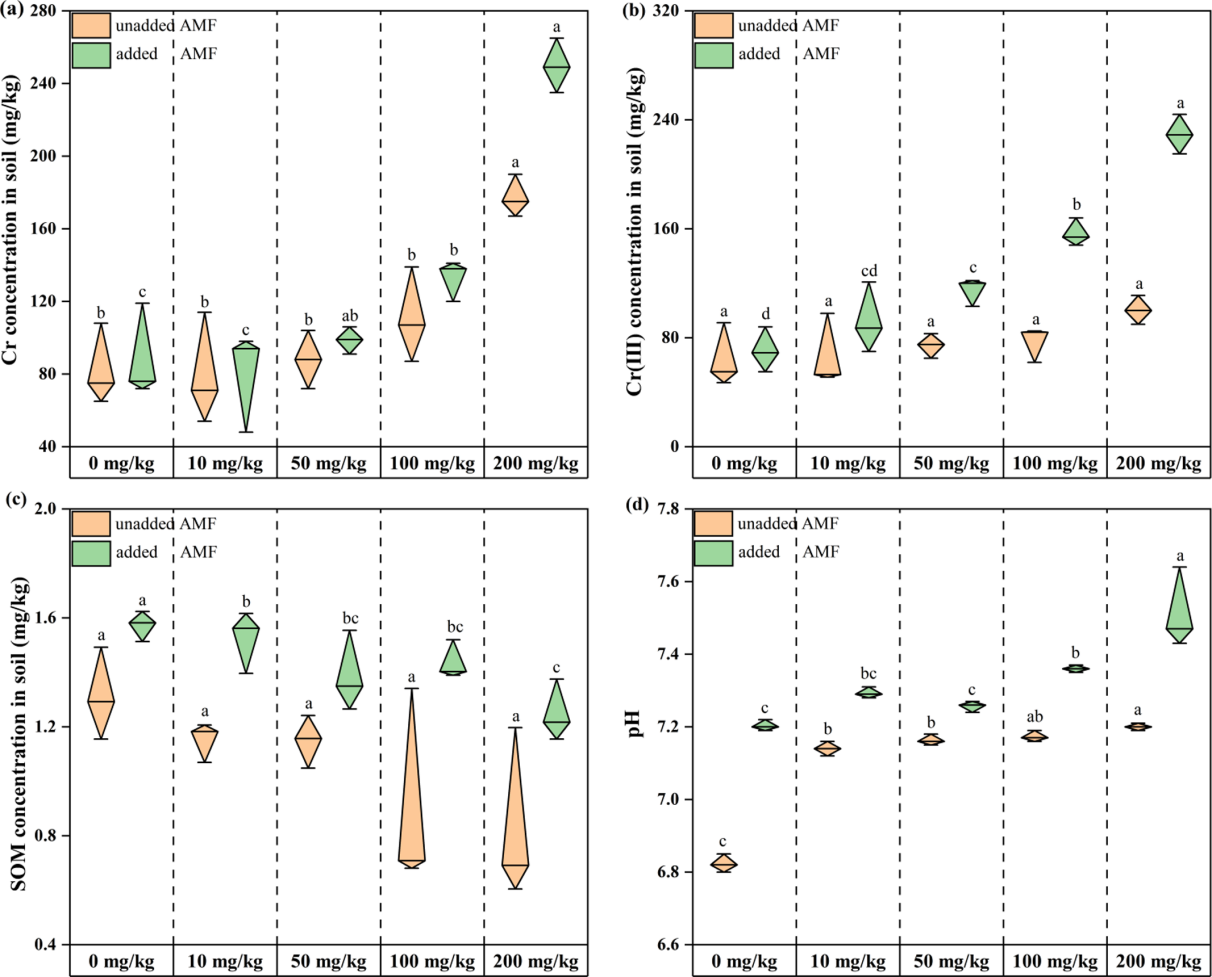
The changes of environmental factors in the rhizosphere soils of *Acorus calamus*. (A) Total Cr, (B) Cr (III) content, (C) SOM content and (D) pH inrhizosphere soils of *Acorus calamus*. Each group of data in the book is obtained by averaging three groups of parallel samples, and different lowercase letters of data in the same column indicate significant differences ( *p* < 0.05).

Total root-soil chromium content increased with increasing Cr concentration throughout the treatment period, and total chromium content in the AMF addition group was higher than in the non-AMF addition group for the same Cr addition level. This difference gradually increased with increasing Cr concentration (6.81%, 9.4%, 10.81%, 16.54%, 28.97% from Group CK to Group D) (Fig. 1A). Soil Cr(III) content showed an increasing trend with increasing Cr(VI) concentration in both groups (Fig. 1B), indicating that Cr(VI) was constantly shifting to Cr(III) in the microenvironment. Root soil Cr(III) levels were increasing, and the shift from Cr(VI) to Cr(III) was facilitated by AMF addition. Root-soil SOM decreased with increasing Cr concentration in both groups, with the lowest SOM in the 200 mg/kg Cr(VI) group, and the highest SOM in the AMF addition group than in the non-AMF addition group at the same concentration (Fig. 1C). The control soil was weakly acidic and after adding potassium dichromate, remained so, and became more alkaline as Cr concentration increased (Fig. 1D). Under Cr (VI) stress, most plants without AMF addition were smaller and had yellow leaves, while plants with added AMF were larger, had more green leaves, and a more developed rhizosphere (Fig. S3).

### Species abundance of fungal community

**Figure 2:**
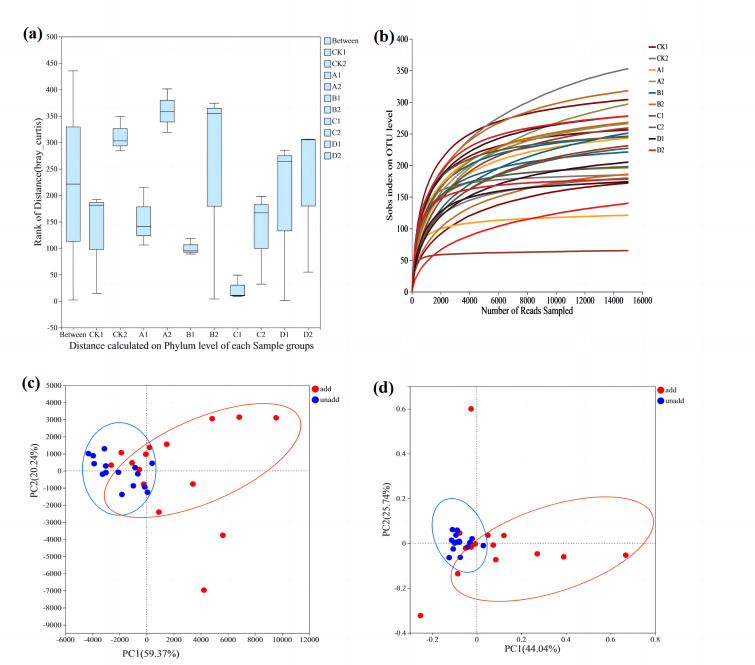
The comparative analysis of the samples. (A) ANOSIM analysis. The *X*-axis is the distance value within or Between groups, the boxes corresponding to between represent the distance value between groups, and the other boxes represent the distance value within groups. The scale on the *Y* axis shows the magnitude of the distance value. It could test whether the difference between groups (two or more groups) is significantly greater than the difference within the group to determine whether the group is meaningful. (B) Dilution curve analysis. The abscissa represents the amount of randomly selected sequencing data. Ordinate, number of species observed. The amount of sequencing data is sufficient according to whether the curve is flat. (C) PCA analysis. The *X*-axis and *Y*-axis represent the two selected principal component axes, and the percentage represents the value of the principal component explaining the difference in sample composition. The red dots represent arbuscular mycorrhizal fungi (AMF) groups and the blue dots represent non-AMF groups. (D) PcoA analysis. The red dots represent AMF groups and the blue dots represent non-AMF groups.

To test whether differences between fungal groups were more significant than those within groups, ANOSIM analysis (Fig. 2A) was conducted using the Bray–Curtis distance algorithm. Between-group differences were considerably more significant than those within groups. Dilution curves after sampling are shown in Fig. 2B, and the amount of data sequenced was sufficient to reach the study level. PCA analysis used to investigate similarities or differences in the composition of the sample groups also showed that the coordinates clearly divided the AMF-added and non-AMF-added groups (Fig. 2D).

**Figure 3:**
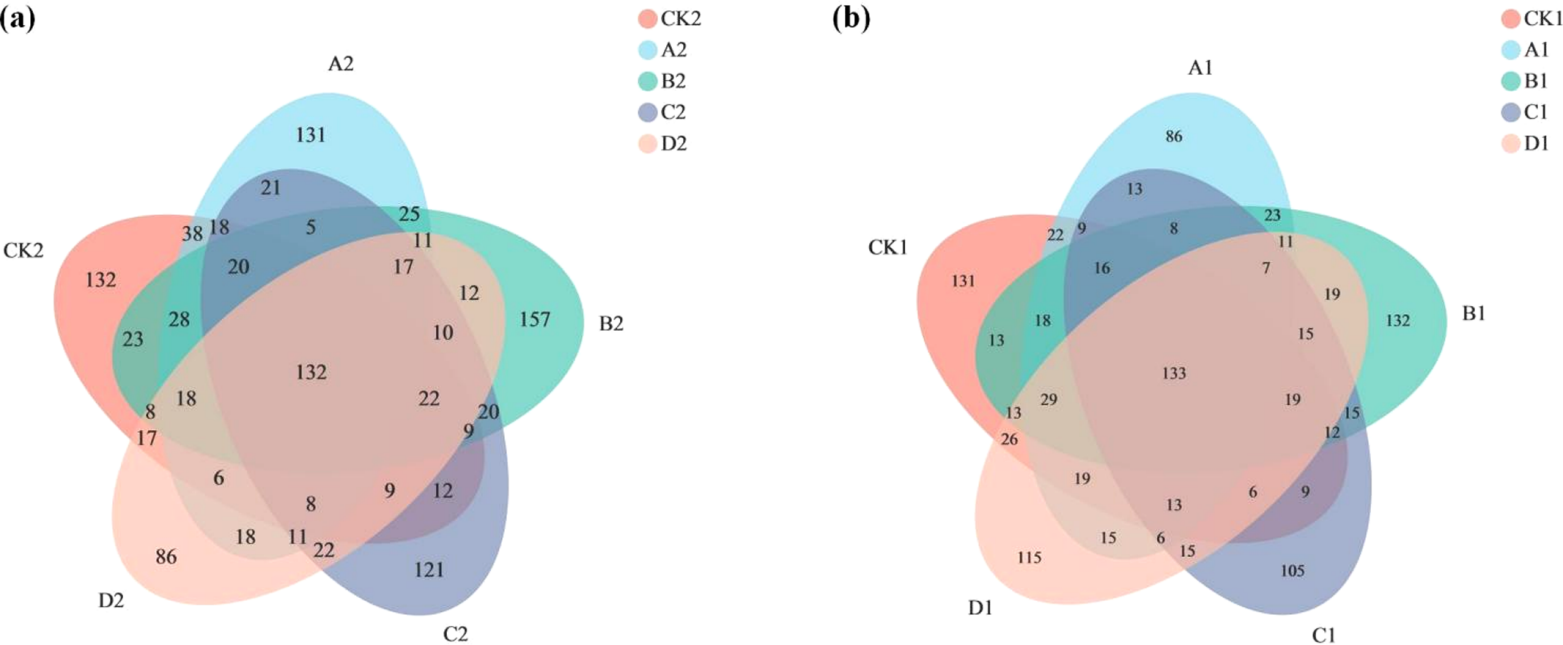
Venn graph. The composition similarity and overlap of species (such as OTU) of environmental samples can be visually displayed. (A) AMF group and (B) non-AMF group.

The Venn diagram shows the number of OTUs common and unique to the added AMF and unadded AMF groups, as well as the similarity of OTU composition and overlap (Fig. 3). In the added AMF group, the number of overlapping OTUs was 132, and in each region the number also varied (Fig. 3A). In the group without the addition of AMF the number of overlapping OTUs was 133, with some variability in the number in each region compared to the group with the addition of AMF (Fig. 3B).

According to the alpha analysis (Table S1) we found that the indices sobs, chao1 and ace, which characterize community richness, varied similarly. Fungal community richness was higher in all groups with AMF than in the control group, but fungal diversity was lower than in the non-AMF group. As Cr(VI) concentration increased, fungal community richness showed an overall decreasing trend, and there were significant differences among different concentrations. Moreover, fungal abundance in the Cr(VI) high concentration group was lower than the control group, and community abundance was lowest when Cr(VI) concentration reached 200 mg/kg. The same pattern can be seen in indices that characterize community diversity such as the Shannon and Simpson, where community diversity was lowest at Cr(VI) concentrations up to 200 mg/kg.

### Fungal community species composition

**Figure 4:**
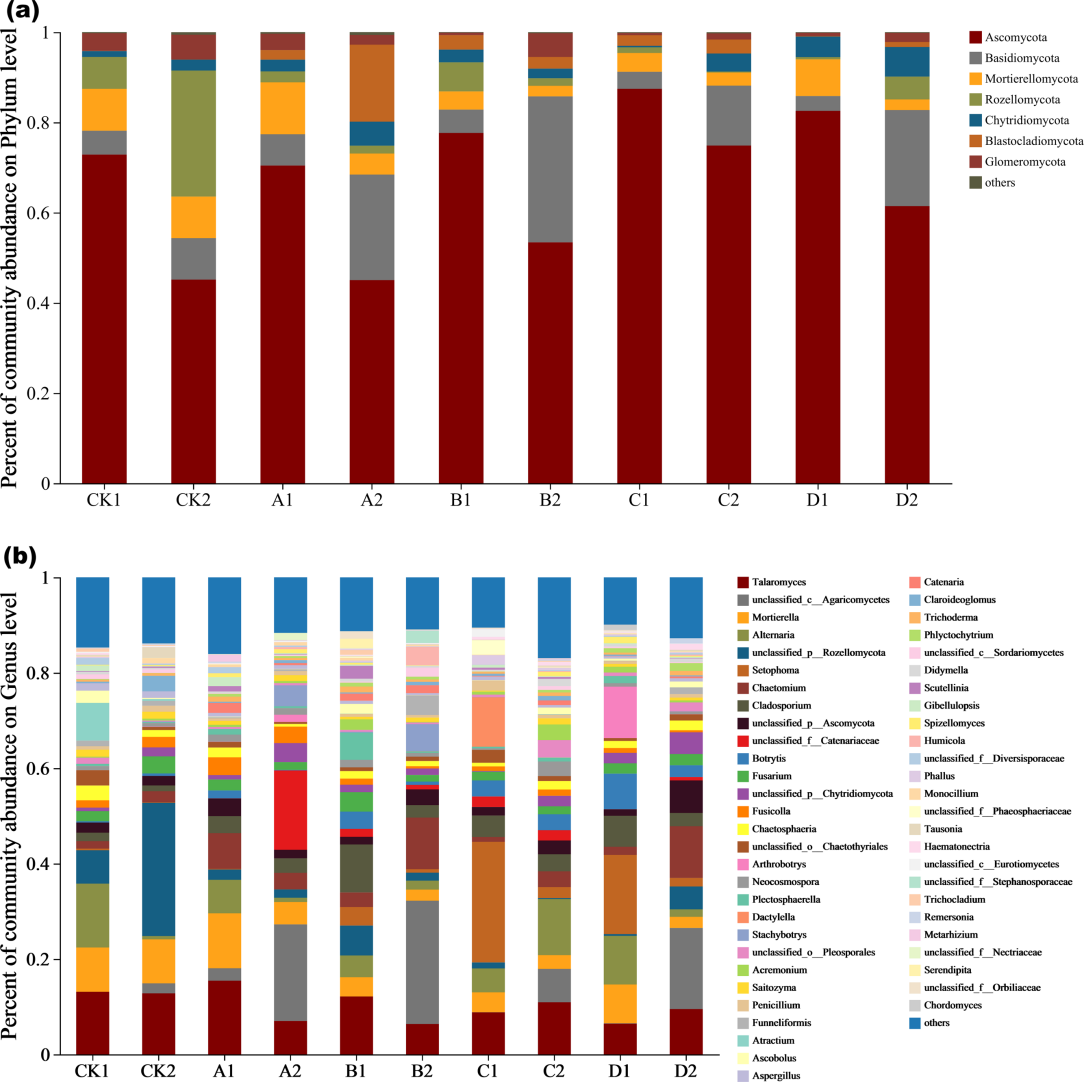
The community plot of the fungal species. (A) Bar diagram of fungal community under phylum classification, (B) bar diagram of fungal community under genus classification.

Fungal species composition at phylum and genus levels differed significantly (Figs. 4A and 4B). The dominant phyla were *Ascomycota*, *Basidiomycota*, *Mortierellomycota*, *Rozellomycota*, *Chytridiomycota*, *Blastocladiomycota*, and *Glomeromycota* (Fig. 4A,). The percentage of dominant phyla in each group varied, but the common denominator was that the dominant species with the highest percentage were *Ascomycota*, accounting for 45.04%-87.43%. *Talaomyces*, *Alternaria*, *Setophoma*, *Cladosporium*, *Fusarium* and *Botrytis* represented *Ascomycota* at the genus level under Cr stress (Fig. 4B). The proportion of *Ascomycota* in the high concentration group (Cr =100–200 mg/kg) treatment was significantly higher than in the low concentration (Cr =50–100 mg/kg) treatment and control. In CK2 (with AMF added), *Rozellomycota* accounted for 27.94% of the dominant species, significantly higher than in the other groups. *Blastocladiomycota* was the most abundant in A2 (AMF added, Cr =10 mg/kg) at 17.06%. Fungal community abundance also differed between AMF treatments at the same Cr concentration, for example, the proportion of *Ascomycota* was significantly higher in the group with AMF addition than in the group without it. *Mortierellomycota* showed characteristics consistent with *Ascomycota*, decreasing in abundance with AMF addition. *Basidiomycota*, however, showed the opposite pattern to the two dominant species, as they increased significantly in the AMF addition group (except for the CK group) in groups A, B, C and D, by 70.31%, 83.99%, 71.51%, and 84.55%, respectively.

**Figure 5:**
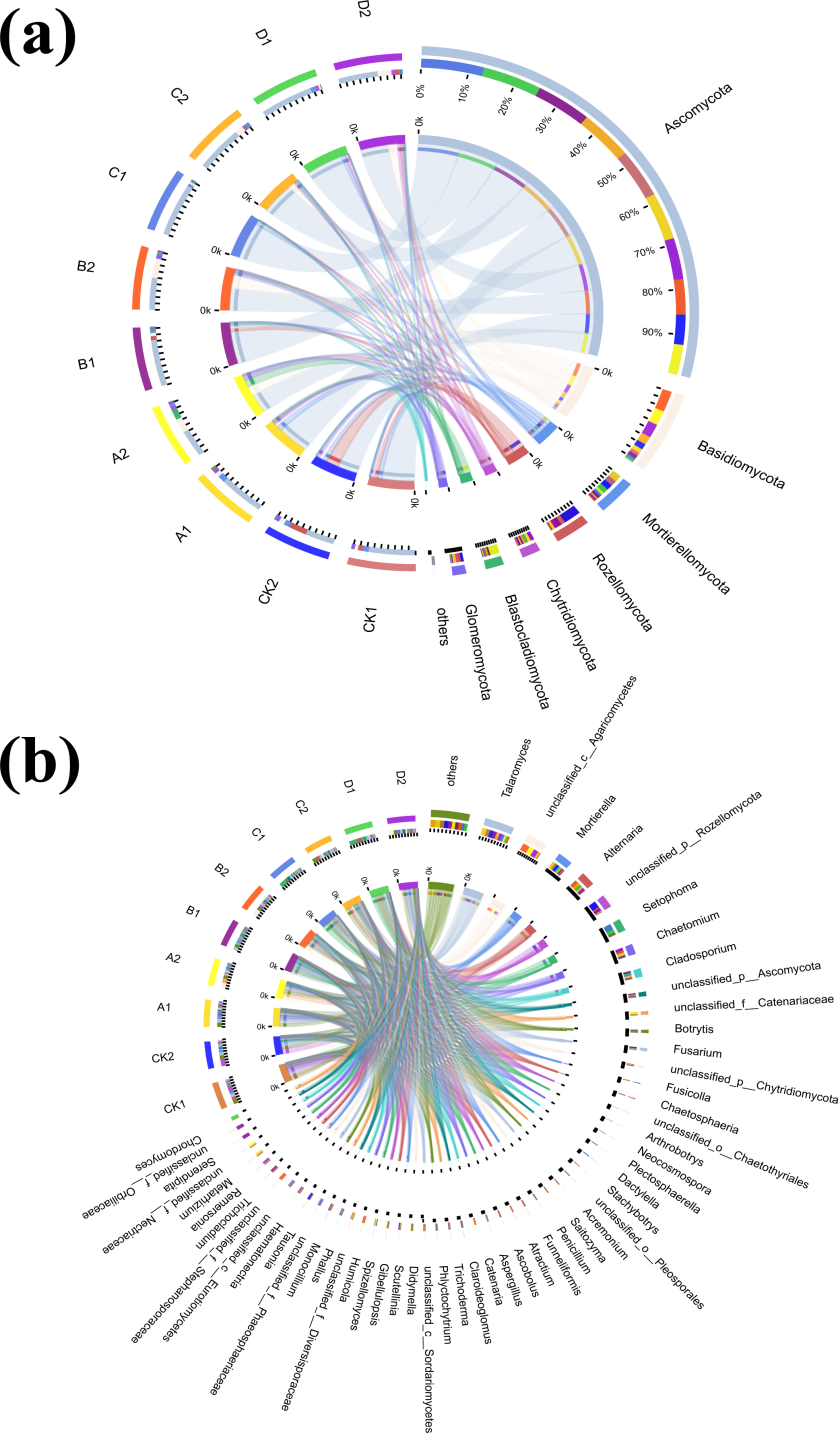
Circos diagram of fungal community. Figure reflected the distribution proportion of dominant species in each sample and the distribution proportion of each dominant species in different samples. (A) Circos diagram of fungal community under phylum classification and (B) genus classification.

To further explore the proportion of different dominant species in each group of samples, as well as the distribution ratio of each dominant species in different subgroups, two circle diagrams (Fig. 5), were constructed to represent fungal species distributions at phylum and genus levels. *Ascomycota* were evenly distributed across treatments, exceeding 70%, and did not significantly change with increasing Cr concentration. However, the proportion of *Ascomycota* in the AMF-added group was lower than at the same Cr concentration in the non-AMF-added group. *Mortierellomycota* had the highest proportion of unspiked A1, followed by a sharp drop in Cr concentration up to 50 mg/kg and then a slight rebound at Cr = 200 mg/kg but not back to the unspiked CK1 *vs.* A1 proportion. *Basidiomycota* was highest in the A1 group (CCr = 10 mg/kg) and then showed a decreasing trend with increasing Cr concentration. *Chytridiomycota* distribution in the different groups fluctuated with increasing Cr concentration, and their percentage decreased sharply when Cr concentration increased from 50 mg/kg to 100 mg/kg and then increased sharply when it reached 200 mg/kg. *Glomeromycota* percentage was highest in the low concentration groups (CK1, A1) and lower in the high concentration groups (B1, C1, D1).

With AMF addition, the proportion of the dominant clade changed significantly. For example, *Mortierellomycota* decreased from the second to the 5th position, while *Blastocladiomycota* increased from last to 4th. *Ascomycota* proportion in each sample group and in different subgroups remained the highest, and increased with increasing Cr concentration until it reached a peak at 100 mg/kg, and then decreased when Cr concentration reached 200 mg/kg. *Rozellomycota* remained the most abundant in the CK2 group, with a significant increase when Cr concentration was 200 mg/kg compared to the non-AMF group, and a significant decrease when Cr concentration was 50 mg/kg. *Blastocladiomycota* distribution characteristics did not change significantly, however, the distribution ratio in different groups increased significantly. The distribution ratio of *Mortierellomycota* reached a maximum in group CK2, and a minimum in B2 (Cr concentration of 50 mg/kg) and D2 (Cr concentration of 200 mg/kg). After AMF addition, the highest *Chytridiomycota* percentage was still found in the D2 group when Cr concentration was 200 mg/kg. Fungal composition also changed significantly at the genus level (Fig. 5B).

### Environmental factor association analysis

**Figure 6:**
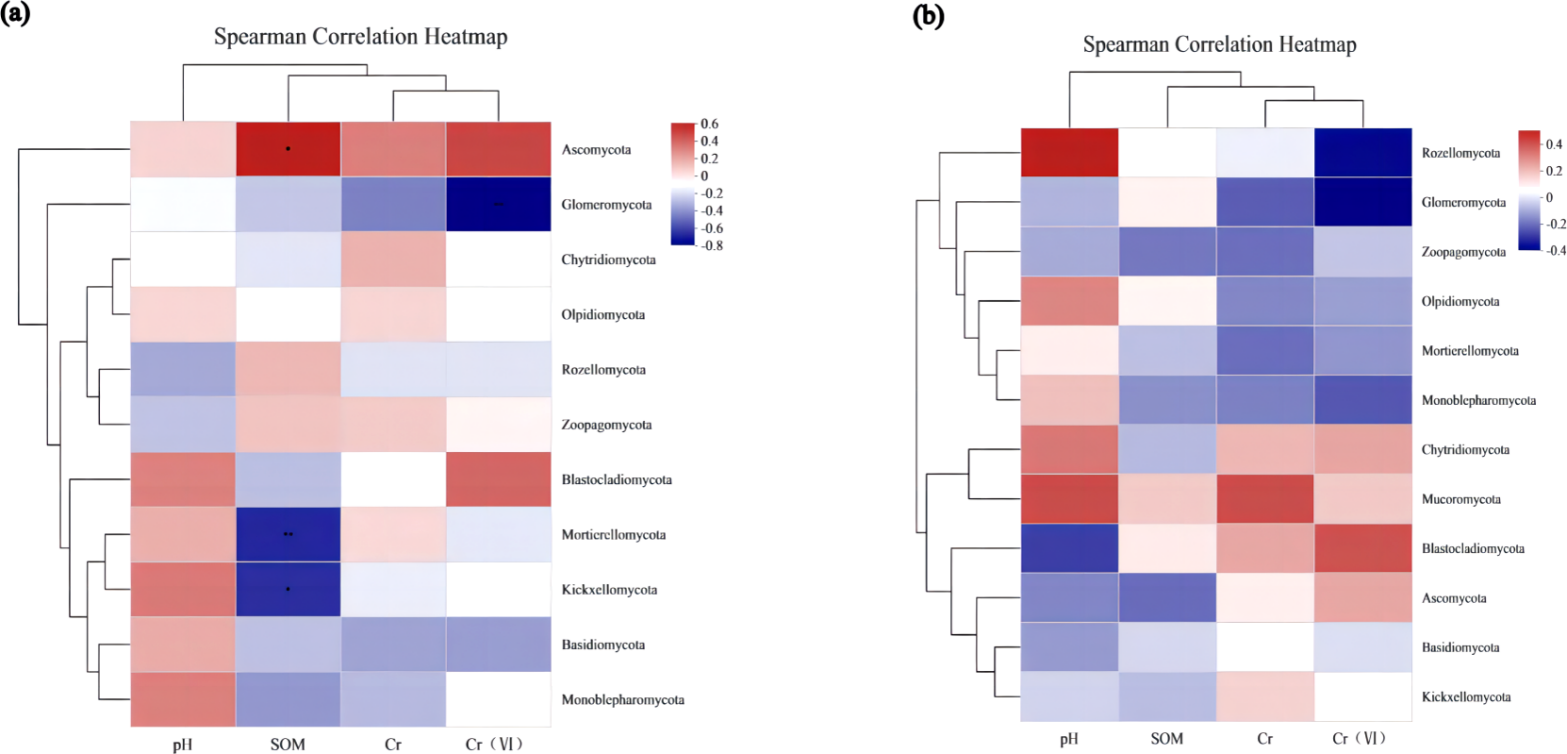
The association analysis diagram of environmental factors. (A) The correlation plots of the measured factors (SOM, pH, Cr, Cr (VI)) and the fungal communities at the phylum level in AMF group; (B) the correlation plots of the measured factors and the fungal communities at the phylum level in the non-AMF group.

**Figure 7:**
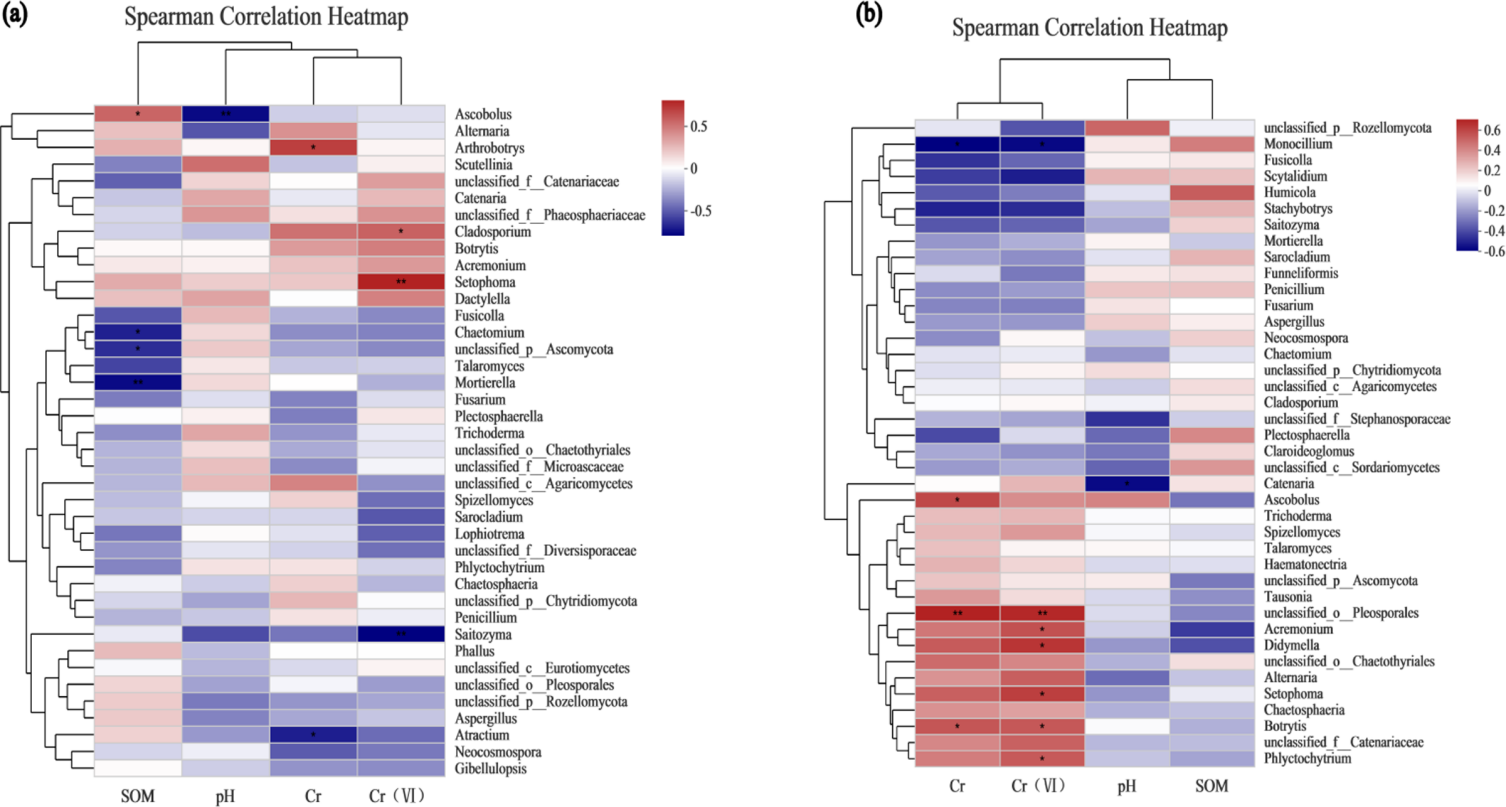
The association analysis diagram of environmental factors. (A) The correlation plots of the measured factors and the fungal communities at the genus level in the AMF group; (B) the correlation plots of the measured factors and the fungal communities at the genus level in the non-AMF group.

To assess the correlation between the soil microenvironment and the structural classification of the fungal community at the phylum and genus level in the AMF addition and control groups, correlation heatmap plots were made based on the spearman analysis index, and these show the top 40 species in terms of total abundance at the genus level (Figs. 6 and 7). Correlations between fungi and pH, SOM, Cr, Cr(VI) at the phylum level changed after AMF addition (Fig. 6). The dominant fungi at the genus level and their correlations with the above factors also changed significantly (Fig. 7A). Of these, Cr(VI) and total Cr correlations with fungal species were significantly altered. Before AMF addition, Cr was negative correlated with *Atractium* (*Ascomycota*) but positively with *Arthrobotrys* (*Ascomycota*) with a correlation coefficient of 0.637 (*p* < 0.05). Similarly, Cr(VI) was negatively correlated with *Saitozyma* (*Basidiomycota*) (*p* < 0.01) but positively correlated with *Setophoma* (*Ascomycota*) (*p* < 0.01), presumably because *Ascomycota* may contain fungi with a corresponding resistance mechanism to Cr stress. After the addition of AMF (Fig. 7B), it was clear that the number of fungi positively correlated with Cr, Cr(VI) increased significantly, with *unclassified Pleosporales* significantly correlated (*p* < 0.01). Cr was positively correlated with *Botrytis* (*Ascomycota*) and *Ascobolus* (*Ascomycota*), and before the addition of AMF showed significant negative correlations with *Ascobolus*, and Cr(VI) showed positive correlations with *Phlyctochytrium* (*Chytridiomycota*), *Didymella* (*Ascomycota*), *Botrytis*, *Setophoma*, and *Acremonium* (*Ascomycota*) showed significant positive correlations, and the change in the correlations revealed that the correlation between some fungi and total chromium changed from negative to positive. Meanwhile there was a significant increase in the number of fungi positively correlated with Cr(VI). Overall it appears that AMF addition: (1) improved Cr resistance of some fungi (mainly *Ascomycota*) to a certain extent, and (2) promoted the ability of fungi to reduce Cr(VI) to Cr(III).

### Functional analysis of fungal community

**Figure 8:**
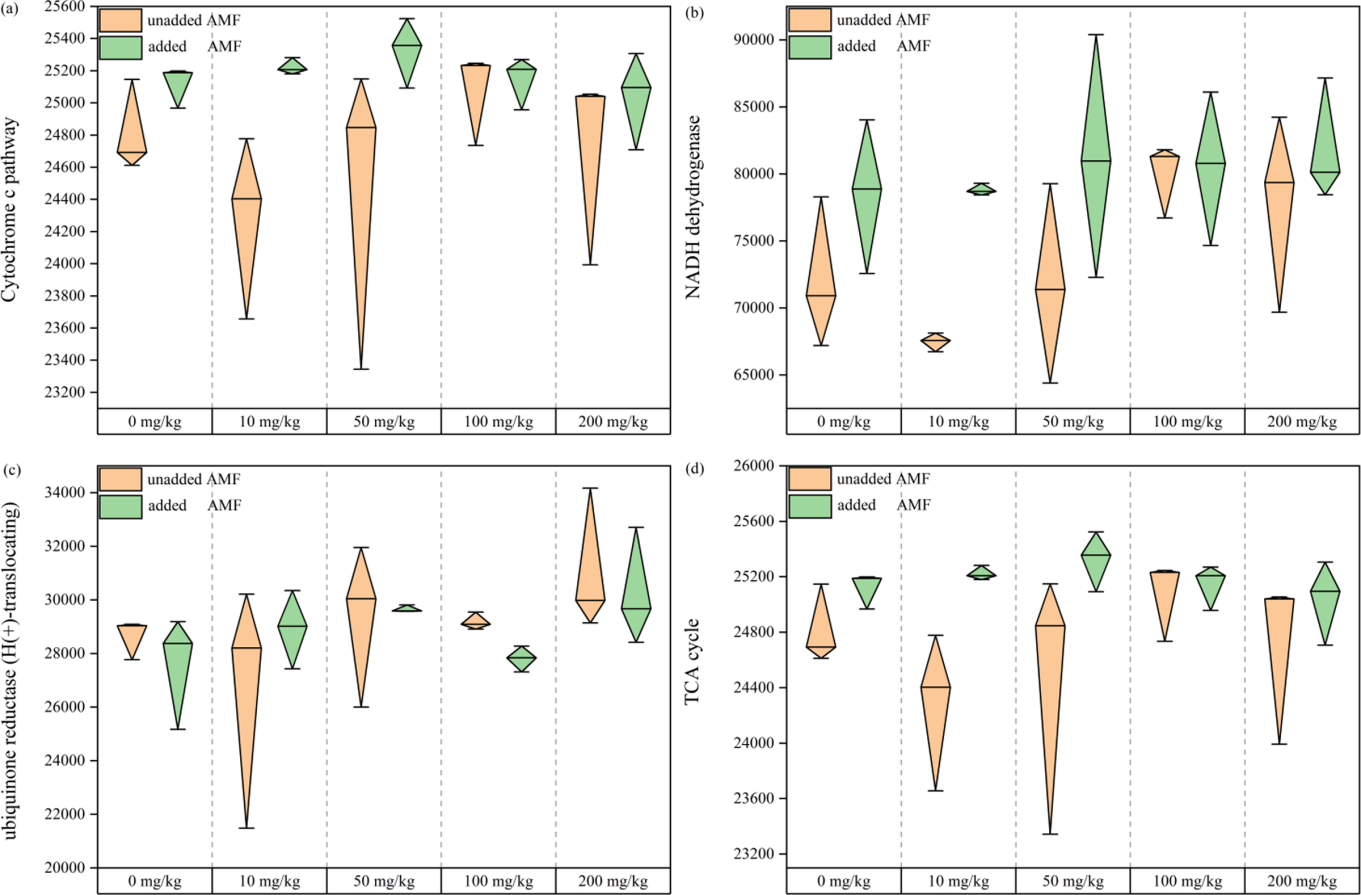
The functional analysis map of fungal community. (A) Cytochrome c pathway; (B) NADH dehydrogenase; (C) NADH: ubiquiinone reductase (H + -translocating); (D) TCA cycle. The *Y*-axis shows the fungal abundance value of each group.

Electrons are transferred from NADH to ubiquinone by the action of dehydrogenase, after which they flow to cytochrome c, and then Cr(VI) receives electrons from cytochrome c to be reduced to Cr(III) (Xia et al., 2018). Fungal abundance values in the cytochrome c pathway showed an overall increase with increasing Cr(VI) concentrations (Fig. 8A), demonstrating that the cytochrome c pathway species increased under Cr stress and became more pronounced with AMF addition. In the AMF group, NADH dehydrogenase fungal abundance increased overall. Species abundance in the NADH dehydrogenase pathway was higher in the AMF group than in the control group (Fig. 8B), and showed an increasing trend in ubiquinone reductase (H(+)-translocating), also more significant in the AMF group than in the control group (Fig. 8C). As an organism essential cycle of energy and material metabolism, TCA uses organic carbon sources (mainly glucose) to produce energy through oxidation by the gluconeogenic pathway. Under Cr(VI) stress, TCA pathway fungal abundance was significantly higher after AMF addition than in the non-AMF group (Fig. 8D). It is hypothesized that the remaining fungal tricarboxylic acid cycle was facilitated by the effect of AMF, with increased use of carbon sources and enhanced energy metabolism.

## Discussion

Under heavy metal stress, microorganisms are more sensitive than other organisms (Charlton et al., 2016; Giller, Witter & McGrath, 2009), and their abundance is altered in various ways. For example, Flavisolbacter and Altererythrobacter abundance is affected by Cr stress (Zhang et al., 2020). Furthermore, fungal community composition was significantly altered in response to changes in heavy metal content (Del Val, Barea & Azcón-Aguilar, 1999; Nordgren et al., 1983). In our study, fungal diversity and abundance were significantly altered by Cr(VI) stress, and overall they showed a decreasing trend with increasing Cr(VI) concentrations in both control and AMF groups. In long-term chronically Cr-contaminated soils, fungal abundance declines but fungal community diversity is not altered (Jin et al., 2018). We showed significant changes in fungal community abundance and diversity at various concentrations of Cr(VI) stress. However, fungal community abundance showed an overall decreasing trend with increasing Cr(VI), especially at high concentrations.

Fungal community species composition changed significantly under Cr stress, and dominant group distribution ratios also changed. The most dominant species under Cr(VI) stress were *Ascomycota*, followed by *Basidiomycota* fungi, and high Cr concentration increased *Ascomycota* fungi. In the correlation heat map, *Ascomycota* fungi were significantly correlated with Cr(VI) and Cr, so it may be that these fungi, along with some in the *Basidiomycota*, are sensitive, or resistant to chromium. *Derxomyces* (*Basidiomycota*) is very sensitive to Cr and shows a significant negative correlation with Cr and *Arthrinium* (Jin et al., 2018). Moreover, some *Ascomycota* fungi can reduce Cr(VI) to Cr(III) using carbon metabolism capabilities, such as *Aspergillus sp*., *Penicillium sp.*, and *Trichoderma hamatum* (Acevedo-Aguilar et al., 2006). Lazarova et al. (2014) found that *Trichosporon* (*Ascomycota*) was able to grow under 10 mmol/LCr stress and was highly resistant to chromium because it can chelate and even reduce Cr(VI) from heavy metal ions (Georgieva et al., 2011; Bajgai, Georgieva & Lazarova, 2012). *Candida* is also highly resistant to Cr and can survive in media containing 100 mmol/L Cr stress due to the presence of chromate reductase (Ramírez-Ramírez et al., 2004). *Aspergillus* (*Ascomycota*) is also a common chromium-resistant fungus that reduces Cr(VI) to Cr(III) (CzakóVér et al., 1999), and responds to Cr contamination mainly by enriching *in vivo* (Mala, Nair & Puvanakrishnan, 2006). Coreno-Alonso et al. (2019) found that the Ed8 strain of *Aspergillus tubingensis* reduced Cr(VI) concentration through reduction reactions stimulated by carboxylic acids and metal chelators.

**Figure 9:**
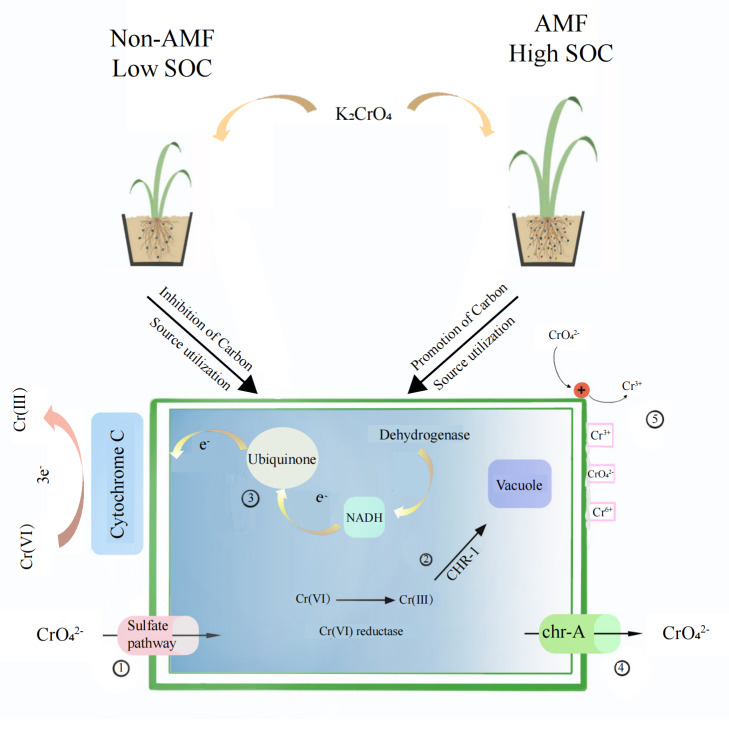
The proposed mechanism of Cr(VI) resistance with the addition of AMF increases the root-soil SOC content and thus fungal activity. (I) Chromate enters through the sulfate channel; (II) Cr(VI) is reduced to Cr(III) through enzymatic and non-enzymatic reduction and subsequently immobilized in the vesicle *via* CHR-1; (III) Cr(VI) is reduced *in vitro* by the fungus through electron transfer; (IV) Chromate is exocytosed through chr-A; (V) The mucus adsorption on the fungal surface, as well as increased protonation levels at low pH, attract chromate ions, which are reduced and then released through electron repulsion release.

Fungal reduction of Cr(VI) reduction can be *in vivo* as well as in vitro(Fig. 9). The conversion of hexavalent chromium to trivalent chromium is through an electron reduction reaction. Microorganisms generally convert hexavalent chromium to trivalent chromium through enzyme and non-enzyme reductions that perform an electron transfer role, transferring 3 electrons to hexavalent chromium. Chromate first enters the fungus through the sulfate pathway due to its similar chemical structure to sulfate, and then a Cr(VI) portion is reduced to Cr(III) through enzymatic and non-enzymatic reduction (non-enzymatic reducing substances such as GSH and cysteine) (Thatoi et al., 2014). Soluble reductases are dominated by ChrR, Yief, and NfoR, and their enzymatic reductions occur under aerobic conditions (Eswaramoorthy et al., 2012; Ackerley et al., 2004; He et al., 2011; Han et al., 2017). The CHR-1 protein, homologous to chrA, is found in a number of *Cysticercus*, *Streptomycetes*, and *Seamycetes* fungi. CHR-1 not only reduces Cr(VI) and immobilizes Cr in fungal vesicles, but chrA is found in many microorganisms as an efflux protein and is an additional measure of resistance to Cr(VI) (Viti et al., 2014).

In addition to the mucus produced by fungi *in vitro* to adsorb heavy metal ions, fungal Cr(VI) reduction *in vitro* is mainly associated with electron transfer. Low pH is also beneficial for Cr(VI) reduction (Martorell et al., 2012). In our study, the soil was weakly acidic, which increases the fungus surface protonation level, making the positively charged surface better able to attract negatively charged chromate ions, which are thereafter reduced to Cr(III) due to electron repulsion release (Park et al., 2005). Xia et al. (2018)) found that Cr(VI) ground electron reduction was achieved by transferring them from NADH to ubiquinone in the presence of dehydrogenase CymA, MtrA, MtrB, MtrC, and OmcA, which are all cytochrome c. We found that Cr(VI) addition added to the fungal cytochrome c metabolic pathway, NADH dehydrogenase, and NADH: ubiquinone reductase, demonstrating that the fungus has *in vitro* reductive effects under Cr(VI) stress.

Fungi are heterotrophic organisms that use soil organic matter as their primary carbon source (Lehmann & Kleber, 2015; Xue et al., 2018), however, their ability to utilize carbon sources is limited at high heavy metal concentrations (Georgieva et al., 2011). However, energy metabolism plays a vital role in the fungal response against heavy metals (Jin et al., 2008). Gasch & Wemer-Washburne (2002) found that many genes involved in carbohydrate and fatty acid metabolism in Common Metal Responsive (CMR) were upregulated in response to heavy metal stress. Moreover, Acevedo-Aguilar et al. (2006) showed that Cr(VI) reduction to Cr(III) by fungi requires a carbon source that is fermented like glucose or oxidized like glycerol, and that total Cr(VI) is unaltered in the absence of a carbon source. AMF can have a beneficial effect on lowering plant exposure to heavy metals by improving water and soil nutrient uptake, increasing aboveground biomass and causing changes in root morphology, reducing oxidative stress from heavy metals (Muhammad et al., 2020). Furthermore, increasing soil SOM with AMF forms fibrous roots with plants, a change that may somewhat improve Cr tolerance of fungal community. The main reason for the increase in soil SOM is that AMF can secrete glycoproteins, such as the globulin-related soil protein (GRSP), which plays a significant role in rhizosphere soil aggregation, carbon storage, and soil quality improvement (Nichols, 2003; Rillig et al., 2001; Schüßler, Schwarzot & Walker, 2001) and can protect AMF mycelia from nutrient loss (Wessels, 1996). AMF and GRSP play crucial roles in direct and indirect soil carbon sequestration. Directly, AMF increases soil carbon content through increased plant growth and aboveground biomass, in the context of root and root deposition input. Indirectly, GRSP improves carbon sequestration (Miller, Reinhardt & Jastrow, 1995) through soil aggregation, forming stable agglomerates that protect organic compounds as well as microbial necromass from enzymatic and microbial attack (Awad et al., 2013). Furthermore, GRSP can increase the active carbon pool by increasing microbial activity (Subramanian et al., 2019; Wright & Upadhyaya, 1998). In our study, AMF addition increased SOM content (Fig. 1C), improved the root system soil microenvironment, improved the limitation of fungal access to carbon sources, increased the fungi metabolic level in the AMF group (Fig. 8D), increased fungal ability to access carbon sources, and increased their own ATP synthesis, which was also beneficial for Cr(VI) reduction.

## Conclusion

Fungal community abundance and diversity were suppressed under Cr stress, especially at high Cr(VI) concentrations (Cr(VI) = 100 mg/kg-200 mg/kg). The dominant species were *Ascomycota*, *Basidiomycota*, and *Mortierellomycota*. Fungi with high tolerance to Cr contamination were present in the *Cysticercus* phylum. After AMF addition, SOM content increased, conversion of Cr(VI) to Cr(III) increased, fungal abundance increased, and fungal diversity increased in the higher concentration groups. The fungal community has a corresponding resistance mechanism under Cr(VI) stress, as Cr(VI) can reduce the metabolic capacity of the fungal community. Cr(VI) reduces root-soil SOM, thus affecting fungal community change, however, AMF increases root-soil SOM content, which also improves the Cr(VI) resistance capacity of fungal community and its ability to enhance Cr(VI) reduction.

Overall, there were significant differences in fungal community changes under different concentrations of Cr (VI), and fungal abundance increased after AMF addition. In contrast, fungal diversity increased at high Cr (VI) concentrations. AMF addition enhanced fungal community Cr(VI) reduction, but the specific fungus responsible and the principal reduction mechanism remain undetermined.

##  Supplemental Information

10.7717/peerj.15681/supp-1 Supplemental Information 1 AMF increases root-soil SOM content, which also improves the Cr(VI) resistance capacity of the fungal community and its ability to enhance Cr(VI) reduction and promote plant growth under Cr (VI) stress

10.7717/peerj.15681/supp-2 Supplemental Information 2 Table S1 *Alpha diversity analysis table* Alpha diversity analysis reflects the richness and diversity of microbial communities, including a series of statistical analysis indexes to estimate the species abundance and diversity of ecological communities.The indexes reflecting the Community richness include: Sobs, Chao1, Ace.The indexes reflecting the Community diversity include: Shannon, Simpson.

10.7717/peerj.15681/supp-3 Supplemental Information 3 * Acorus calamus* pot experiment design Experimental groups were divided into AMF-added, and non-AMF-added, each with five Cr levels: 0, 10, 50, 100, 200 mg/kg respectively corresponded to CK, A, B, C, D group, and three parallel groups for each treatment.

10.7717/peerj.15681/supp-4 Supplemental Information 4 AMF colonization map Successful colonization of arbuscular mycorrhizal fungi under electron microscope

10.7717/peerj.15681/supp-5 Supplemental Information 5 Plant growth map Comparison of growth of *Acorus calamus* under Cr stress. Left:the plants without the addition of AMF; Right:the plants with the addition of AMF

10.7717/peerj.15681/supp-6 Supplemental Information 6 The raw data for soil properties

## Additional Information and Declarations

### Data Availability

The following information was supplied regarding data availability: The sequence reads are available at BioProject: PRJNA952550.

## References

[ref-1] Acevedo-Aguilar FJ, Espino-Saldana AE, Leon-Rodriguez IL, Rivera-Cano ME, vila Rodriguez M (2006). Hexavalentchromium removal in vitro and from industrial wastes, using chromate-resistant strains of filamentous fungi indigenous to contaminated wastes. Canadian Journal of Microbiology.

[ref-2] Ackerley DF, Gonzalez CF, Park CH, Blake R, Keyhan M, Matin A (2004). Chromate-reducing properties of soluble flavoproteins from *Pseudomonas putida* and *Escherichia coli*. Applied and Environmental Microbiology.

[ref-3] Aly AH, Debbab A, Proksch P (2011). Fungal endophytes: unique plant inhabitants with great promises. Applied Microbiology and Biotechnology.

[ref-4] Ammara B, Saleh TA (2020). Removal of toxic metals from wastewater in constructed wetlands as a green technology; catalyst role of substrates and chelators. Ecotoxicology and Environmental Safety.

[ref-5] Awad YM, Blagodatskaya E, Ok YS, Kuzyakov Y (2013). Effects of polyacrylamide, biopolymer and biochar on the decomposition of 14C-labelled maize residues and on their stabilization in soil aggregates. European Journal of Soil Science.

[ref-6] Bajgai RC, Georgieva N, Lazarova N (2012). Bioremediation of chromium ions with filamentous yeast Trichosporon cutaneum R57. Journal of Biology and Earth Sciences.

[ref-7] Balestrini R, Chitarra W, Antoniou C, Ruocco M, Fotopoulos V (2018). Improvement of plant performance under water deficit with the employment of biological and chemical priming agents. The Journal of Agricultural Science.

[ref-8] Bartlett RJ (1991). Chromium cycling in soils and water –links, gaps, and methods. Environmental Health Perspectives.

[ref-9] Brundrett MC, Tedersoo L (2018). Evolutionary history of mycorrhizal symbioses and global host plant diversity. New Phytologist.

[ref-10] Calheiros CSC, Pereira SIA, Franco AR, Castro PML (2019). Diverse Arbuscular mycorrhizal fungi (AMF) communities colonize plants inhabiting a constructed wetland for wastewater treatment. Water.

[ref-11] Charlton A, Sakrabani R, Tyrrel S, Casado MR, McGrath SP, Crooks B, Cooper P, Campbell CD (2016). Long-term impact of sewage sludge application on soil microbial biomass: an evaluation using meta-analysis. Environmental Pollution.

[ref-12] Chen BF, Huang S, Liu B, Ge Q, Xie SS (2018a). Thermodynamic analysis for separation of vanadium and chromium inV(IV)-Cr(III)-H2O system. Transactions of Nonferrous Metals Society of China.

[ref-13] Chen BS, Teh B, Sun C, Hu SR, Lu XM, Boland W, Shao YQ (2016). Biodiversity and activity of the gut microbiota across the life history of the insect herbivore Spodoptera littoralis. Scientific Reports.

[ref-14] Chen SF, Zhou YQ, Chen YR, Gu J (2018b). fastp: an ultra-fast all-in-one FASTQ preprocessor. Bioinformatics.

[ref-15] Coreno-Alonso A, Acevedo-Aguilar FJ, Reyna-Lopez GE, Tomasini A, Fernandez FJ, Wrobel K (2019). Diverse Arbuscular Mycorrhizal Fungi (AMF) communities colonize plants inhabiting a constructed wetland for wastewater treatment. Water.

[ref-16] CzakóVér K, Batiè M, Raspor P, Sipiczki M, Pesti M (1999). Hexavalent chromium uptake by sensitive and tolerant mutants of Schizosaccharomyces pombe. FEMS Microbiology Letters.

[ref-17] Del Val C, Barea JM, Azcón-Aguilar C (1999). Diversity of arbuscular mycorrhizal fungus populations in heavy-metal-contaminated soils. Applied and Environmental Microbiology.

[ref-18] Devi TS, Gupta S, Kapoor R (2019). Arbuscular mycorrhizal fungi in alleviation of cold stress in plants. Advancing frontiers in mycology & mycotechnology.

[ref-19] Dhal B, Thatoi HN, Das NN, Pandey BD (2013). Chemical and microbial remediation of hexavalent chromium from contaminated soil and mining/metallurgical solid waste: a review. Journal of Hazardous Materials.

[ref-20] Eswaramoorthy S, Poulain S, Hienerwadel R, Bremond N, Sylvester MD, Zhang YB (2012). Crystal structure of ChrR–a quinone reductase with the capacity to reduce chromate. PLOS ONE.

[ref-21] Fan MC, Xiao X, Guo YQ, Zhang J, Wang ET, Chen WM, Lin YB, Wei GH (2017). Thirty-one years of rice-rice-green manure rotations shape the rhizosphere microbial community and enrich beneficial bacteria. Soil Biology and Biochemistry.

[ref-22] Garcıa-Hernández MA, Villarreal-Chiu JF, Garza-González MT (2017). Metallophilic fungi research: an alternative for its use in the bioremediation of hexavalent chromium. International Journal of Environmental Science and Technology.

[ref-23] Gasch AP, Wemer-Washburne M (2002). The genomics ofyeast responses to environmental stress and starvation. Functional & Integrative Genomics.

[ref-24] Georgieva N, Peshev D, Rangelova N, Lazarova N (2011). Effect of hexavalent chromium on growth of Trichosporon cutaneum R57. Journal of Chemical Technology and Metallurgy.

[ref-25] Giller KE, Witter E, McGrath SP (2009). Heavy metals and soil microbes. Soil Biology and Biochemistry.

[ref-26] Hall JL (2002). Cellular mechanisms for heavy metal detoxification and tolerance. Journal of Experimental Botany.

[ref-27] Han H, Ling Z, Zhou T, Xu R, He Y, Liu P, Li X (2017). Copper (II) binding of NAD(P)H- flavin oxidoreductase (NfoR) enhances its Cr (VI)-reducing ability. Scientific Reports.

[ref-28] He M, Li X, Liu H, Mille SJ, Wang G, Rensing C (2011). Characterization and genomic analysis of a highly chromate resistant and reducing bacterial strain Lysinibacillus fusiformis ZC1. Journal of Hazardous Materials.

[ref-29] Hu SS, Hu B, Chen ZB, Vosátka M, Vymazal J (2021). Arbuscular mycorrhizal fungi modulate the chromium distribution and bioavailability in semi-aquatic habitats. Chemical Engineering Journal.

[ref-30] Jan N, Lerika K, Jaroslav S (2009). Trace elements in Phragmites australis growing in constructed wetlands for treatment of municipal wastewater. Ecological Engineering: The Journal of Ecotechnology.

[ref-31] Janoušková M, Pavlíková D, Vosátka M (2006). Potential contribution of arbuscular mycorrhiza to cadmium immobilisation in soil. Chemosphere.

[ref-32] Jin HU, Meng DL, Liu XD, Liang YL, Yin HQ, Liu HW (2018). Response of soil fungal community to long-term chromium contamination. Transactions of Nonferrous Metals Society of China.

[ref-33] Jin YH, Dunlap PE, McBride SJ, Al-Refai H, Bushel PR, Freedman JH (2008). Global transcriptome and deletome profiles of yeast exposed to transition metals. PLOS Genetics.

[ref-34] Joutey NT, Sayel H, Bahafid W, El Ghachtouli N (2015). Mechanisms of hexavalent chromium resistance and removal by microorganisms. Reviews of Environmental Contamination and Toxicology.

[ref-35] Lazarova N, Krumova E, Stefanova T, Georgieva N, Angelova M (2014). The oxidative stress response of the filamentous yeast Trichosporon cutaneum R57 to copper, cadmium and chromium exposure. Biotechnology and Biotechnological Equipment.

[ref-36] Lehmann J, Kleber M (2015). The contentious nature of soil organic matter. Nature.

[ref-37] Lenoir I, Fontaine J, Sahraoui AL-H (2016). Arbuscular mycorrhizal fungal responses to abiotic stresses: a review. Phytochemistry.

[ref-38] Li C, Zhou K, Qin W, Tian C, Qi M (2019a). A review on Heavy metals contamination in soil: effects, sources, and remediation techniques. Soil and Sediment Contamination.

[ref-39] Lin J, Wang Y, Sun S, Mu C, Yan X (2017). Effects of arbuscular mycorrhizal fungi on the growth, photosynthesis and photosynthetic pigments of Leymus chinensis seedlings under salt-alkali stress and nitrogen deposition. Science of The Total Environment.

[ref-40] Mala JGS, Nair BU, Puvanakrishnan R (2006). Bioaccumulation and biosorption of chromium by Aspergillus niger MTCC 2594. Journal of General & Applied Microbiology.

[ref-41] Manikandan NA, Alemu AK, Goswami L, Pakshirajan K, Pugazhenthi G (2016). Waste litchi peels for Cr(VI) removal from synthetic wastewater in batch and continuous systems: sorbent characterization, regeneration and reuse study. Journal of Environmental Engineering.

[ref-42] Martorell MM, Fernandez PM, Farina JI, Figueroa L (2012). Cr(VI) reduction by cell-free extracts of Pichia jadinii and Pichia anomala isolated from textile-dye factory effluents. International Biodeterioration & Biodegradation.

[ref-43] Miller R, Reinhardt D, Jastrow J (1995). External hyphal production of vesiculararbuscular mycorrhizal fungi in pasture and tallgrass prairie communities. Oecologia.

[ref-44] Muhammad R, Muhammad K, Yizeng F, Wang XR (2020). Arbuscular mycorrhizal fungi-induced mitigation of heavy metal phytotoxicity in metal contaminated soils: a critical review. Journal of Hazardous Materials.

[ref-45] Nataša D, Gaberščik A (2010). Mycorrhizal colonization and growth of Phragmites australis in an intermittent wetland. Aquatic Botany.

[ref-46] Nichols KA (2003). Characterization of Glomalin, a Glycoprotein Produced by Arbuscular Mycorrhizal Fungi, Coll. Agric. Nat. Resour. Univ. Maryl. University of Maryland.

[ref-47] Nordgren A, Baath E, Soderstrom B (1983). Microfungi and microbial activity along a heavy metal gradient. Applied and Environmental Microbiology.

[ref-48] Park D, Yun YS, Jo JH, Park JM (2005). Mechanism of hexavalent chromium removal by dead fungal biomass of Aspergillus niger. Water Research.

[ref-49] Piyush M, Singh A, Anderson TA (2020). Aquatic phytoremediation strategies for chromium removal. Reviews in Environmental Science and Bio/Technology.

[ref-50] R Core Team (2016). https://www.r-project.org.

[ref-51] Redecker D, Schüßler A, Stockinger H, Stürmer SL, Morton JB, Walker C (2013). An evidence-based consensus for the classification of arbuscular mycorrhizal fungi (*Glomeromycota*). Mycorrhiza.

[ref-52] Rillig MC, Wright SF, Nichols KA, Schmidt WF, Torn MS (2001). Large contributionof arbuscular mycorrhizal fungi to soil carbon pools in tropical forest soils. Plant Soil.

[ref-53] Ramírez-Ramírez R, Calvo-Méndez C, Avila-Rodríguez M, Lappe P, Ulloa M, Vázquez-Juárez R (2004). Cr(VI) reduction in a chromate-resistant strain of Candida maltosa isolated from the leather industry. Antonie van Leeuwenhoek.

[ref-54] Schüßler A, Schwarzot D, Walker C (2001). A new fungal phylum, the *Glomeromycota*: phylogeny and evolution. Mycological Research.

[ref-55] Shi L, Xue JW, Liu BH, Dong PC, Wen ZG, Shen ZG, Chen YH (2018). Hydrogen ions and organic acids secreted by ectomycorrhizal fungi, Pisolithus Pisolithus sp. 1 LS-2017, are involved in the efficient removal of hexavalent chromium from waste water. Ecotoxicology and Environmental Safety.

[ref-56] Subramanian KS, Vivek PN, Balakrishnan N, Nandakumar NB, Rajkishore SK (2019). Effects of arbuscular mycorrhizal fungus rhizoglomus intraradices on active and passive pools of carbon in long-term soil fertility gradients of maize based cropping system. Archives of Agronomy and Soil Science.

[ref-57] Sultana MY, Akratos CS, Pavlou S, Vayenas DV (2014). Chromium removal in constructed wetlands: a review. International Biodeterioration & Biodegradation.

[ref-58] Thatoi H, Das S, Mishra J, Rath BP, Das N (2014). Bacterial chromate reductase, a potential enzyme for bioremediation of hexavalent chromium: a review. Journal of Environmental Management.

[ref-59] Torres N, Antolín MC, Goicoechea N (2018). Arbuscular mycorrhizal symbiosis as a promising resource for improving berry quality in grapevines under changing environments. Frontiers in Plant Science.

[ref-60] Viti C, Giovannetti L, Singh SN, Tripathi RD (2007). Bioremediation of soils pollutedwith hexavalent chromium using bacteria-the challenge. Environmental bioremediation technologies.

[ref-61] Viti C, Marchi E, Decorosi F, Giovannetti L (2014). Molecular mechanisms of Cr(VI) resistance in bacteria and fungi. FEMS Microbiology Letters.

[ref-62] Vodnik D, Grcman H, Macek I, van Elteren JT, Kovacevic M (2008). The contribution of glomalin-related soil protein to Pb and Zn sequestration in polluted soil. Science of the Total Environment.

[ref-63] Wang BC, Zhu SX, Li WJ, Tang Q, Luo YH (2021). Effects of chromium stress on the rhizosphere microbial community composition of Cyperus alternifolius. Ecotoxicology and Environmental Safety.

[ref-64] Wang Q, Huang L, Pan Y, Quan X, Puma GLi (2017a). Impact of Fe(III) as an effective electron-shuttle mediator for enhanced Cr(VI) reduction in microbial fuel cells: reduction of diffusional resistances and cathode overpotentials. Journal of Hazardous Materials.

[ref-65] Wang Y, Su H, Gu Y, Song X, Zhao J (2017b). Carcinogenicity of chromium and chemoprevention: a brief update. OncoTargets and Therapy.

[ref-66] Wang Y, Wang M, Li Y, Wu A, Huang J (2018). Effects of arbuscular mycorrhizal fungi on growth and nitrogen uptake of Chrysanthemum morifolium under salt stress. PLOS ONE.

[ref-67] Wessels JGH (1996). Hydrophobins: proteins that change the nature of the fungal surface. Advances in Microbial Physiology.

[ref-68] Wright SF, Upadhyaya A (1998). A survey of soils for aggregate stability and glomalin, aglycoprotein produced by hyphae of arbuscular mycorrhizal fungi. Plant Soil.

[ref-69] Xia SP, Song ZL, Jeyakumar P, Shaheen SM, Rinklebe J, OKY S, Bolan N, Wang HL (2019). A critical review on bioremediation technologies for Cr(VI)-contaminated soils and wastewater. Critical Reviews in Environmental Science and Technology.

[ref-70] Xia X, Wu S, Li N, Wang D, Zheng S, Wang G (2018). Novel bacterial selenite reductase CsrF responsible for Se(IV) and Cr(VI) reduction that produces nanoparticles in Alishewanella sp. WH16-1. Journal of Hazardous Materials.

[ref-71] Xue SG, Li M, Jiang J, Millar GJ, Li CX, Kong XF (2018). Phosphogypsum stabilization of bauxite residue: conversion of its alkaline characteristics. Journal of Environmental Sciences.

[ref-72] Xue WJ, Huang DL, Zeng GM, Wan J, Cheng M, Zhang C, Hu CJ, Li J (2018b). Performance and toxicity assessment of nanoscale zero valent iron particles in the remediation of contaminated soil: a review. Chemosphere.

[ref-73] Xue WJ, Peng, W Z, Huang DL, Zeng, M G, Wan J, Xu R, Cheng M, Zhang C, Jiang D, Hu Z (2018a). Nanoremediation of cadmium contaminated river sediments: microbial response and organic carbon changes. Journal of Hazardous Materials.

[ref-74] Xue XM, Yan Y, Xu HJ, Wang N, Zhang X, Ye J (2014). ArsH from Synechocystis sp. PCC 6803 reduces chromate and ferric iron. FEMS Microbiology Letters.

[ref-75] Zhan FD, Li B, Jiang M, Li TG, He YM, Li Y, Wang YS (2019). Effects of arbuscular mycorrhizal fungi on the growth and heavy metal accumulation of bermudagrass [Cynodon dactylon (L.) Pers.] grown in a lead-zinc mine wasteland. International Journal of Phytoremediation.

[ref-76] Zhang SY, Hao XD, Tang JH, Hu J, Deng Y, Xu ML, Zhu P, Tao JM, Liang YL, Yin HG, Jiang LH, Liu XD, Liu HW (2020). Assessing chromium contamination in red soil: monitoring the migration of fractions and the change of related microorganisms. International Journal of Environmental Research and Public Health.

[ref-77] Zhao W, Chen ZB, Yang XQ, Zhu SX (2023). Integrated transcriptomics and metabolomics reveal key metabolic pathway responses in Pistia stratiotes under Cd stress. Journal of Hazardous Materials.

