# Supplementary figures and images for: Arbuscular mycorrhizal fungi alter rhizosphere fungal community characteristics of Acorus calamus to improve Cr resistance
Source: PeerJ. 2023 Nov 8;11:e15681. doi: 10.7717/peerj.15681 (PMC10638908; doi:10.7717/peerj.15681)

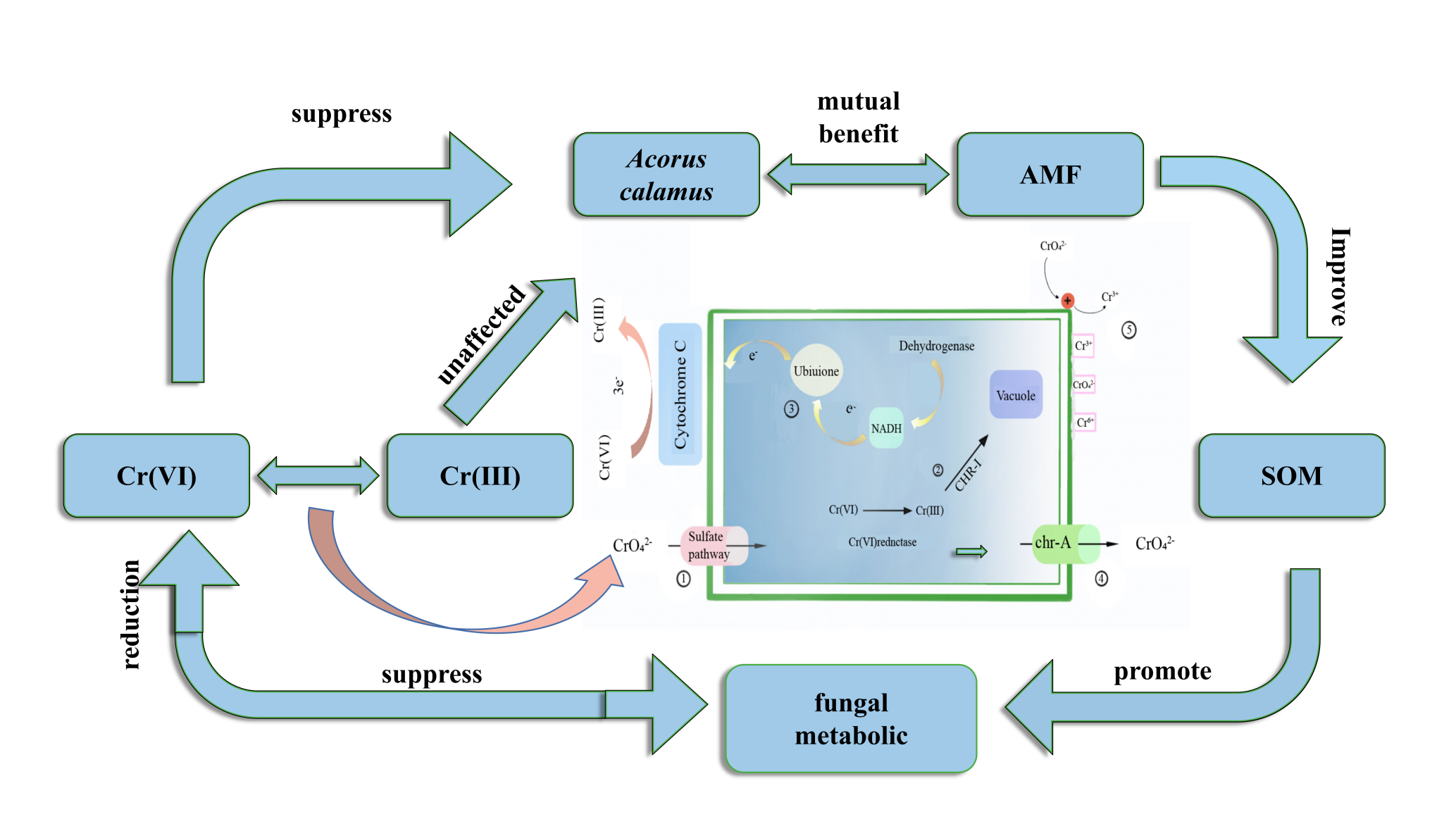

Supplement: Supplemental Information 1 [file peerj-11-15681-s001.png]

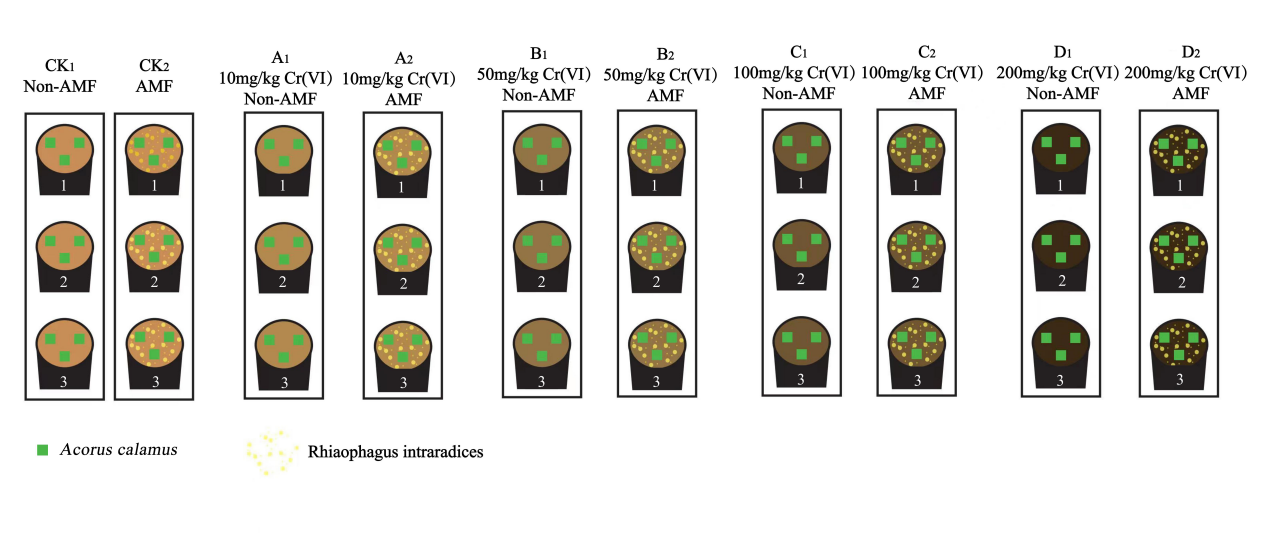

Supplement: Supplemental Information 3 — Experimental groups were divided into AMF-added, and non-AMF-added, each with five Cr levels: 0, 10, 50, 100, 200 mg/kg respectively corresponded to CK, A, B, C, D group, and three parallel groups for each treatment. [file peerj-11-15681-s003.png]

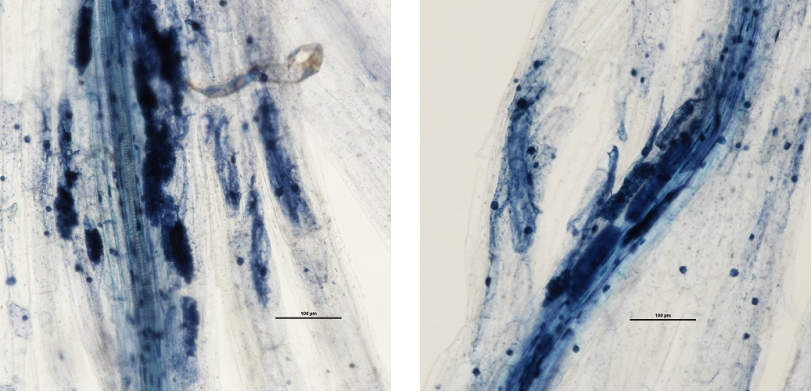

Supplement: Supplemental Information 4 — Successful colonization of arbuscular mycorrhizal fungi under electron microscope [file peerj-11-15681-s004.png]

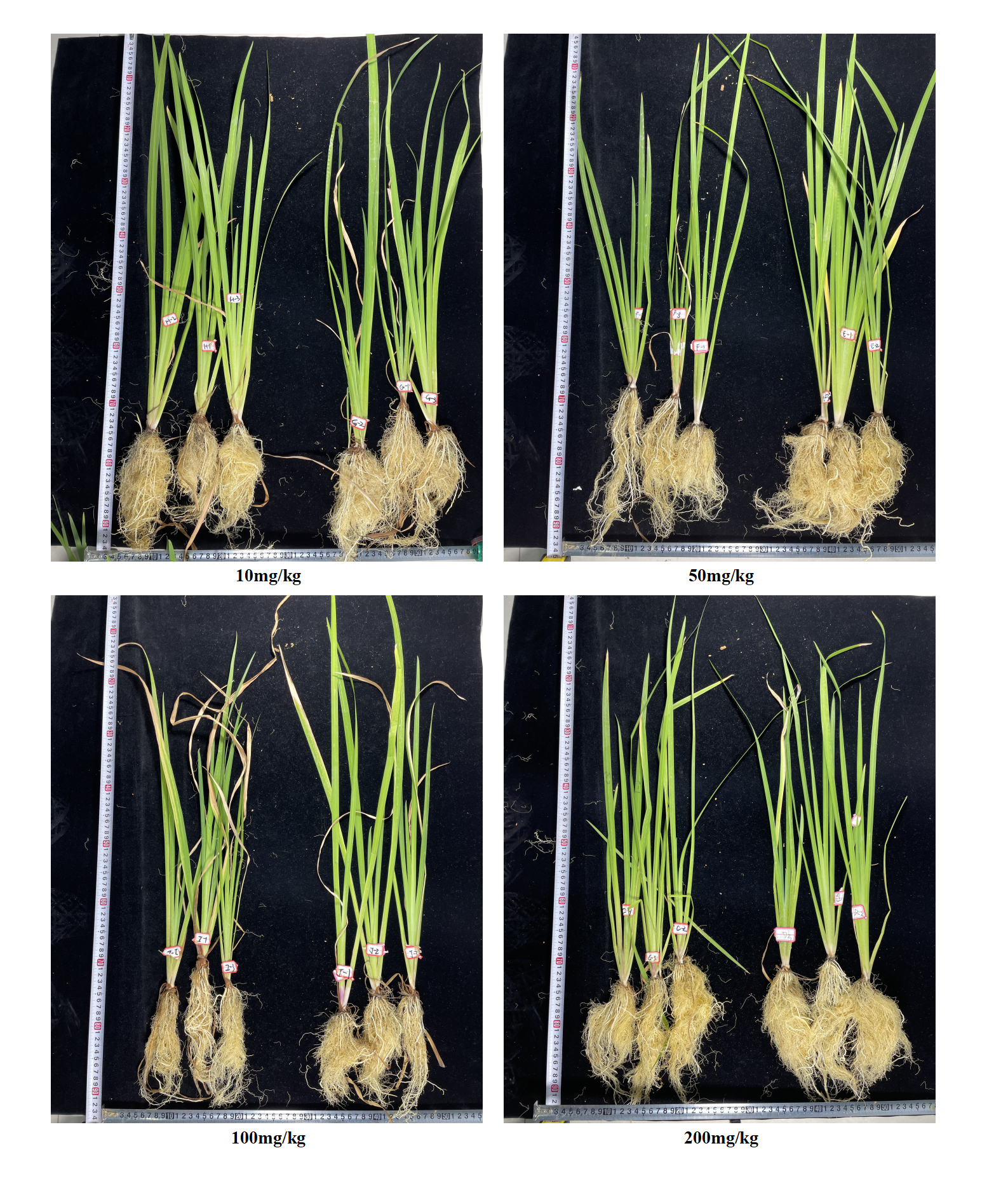

Supplement: Supplemental Information 5 — Comparison of growth of Acorus calamus under Cr stress. Left:the plants without the addition of AMF; Right:the plants with the addition of AMF [file peerj-11-15681-s005.png]
